# Diffuse Hæmatocele of the Spermatic Cord

**Published:** 1853-05

**Authors:** 


*Diffuse Hæmatocele of the Spermatic Cord.*—Under the above title we are desirous of placing together the particulars of two very interesting cases, examples of an affection of very infrequent occurrence, and involving some important practical points.

CASE I. *Kick on the Groin.—Large Extravasation of Blood into the structure of the cord.—Expectant treatment.—Slow absorption of the effused blood.* Under the care of Mr. STANLEY.—John Davis, aged 30, a tall, powerful man, was admitted on Dec. 15, 1851, on account of a large swelling on the left side of his scrotum. The tumor was the size of two fists, pyriform in shape, its neck extending into the inguinal canal, very tense, with a smooth rounded outline, and equally solid feeling in all parts. It completely concealed the testicle and cord. The skin of the scrotum was distended, and of a purplish-red hue; it did not, however, adhere to the swelling, but might be pinched up from it. The man stated that he had on the previous day received a violent kick from a horse in the left groin, immediately after which swelling of the purse commenced, and, continuing to increase, attained, in the course of six hours, its present size. The symptoms and history agreeing so well, Mr. Stanley felt no difficulty in diagnosing it as a case of extravasation of blood into the tissues of the spermatic cord, and he accordingly ordered the patient to be confined to the recumbent posture, and to keep a cold lotion constantly applied to the part. About ten days later, and before any apparent diminution in the size of the swelling had taken place, it was discovered that in the anterior and lower part there was a soft spot, and this, in the course of a few days, had become much more plainly evident. The sensation given to the finger was so like that of fluctuation, that several times the question of the propriety of making a puncture into it was mooted. Its performance was, however, deferred, and the swelling gradually diminishing in size from below upwards, it became evident in a short time that this false sense of fluctuation had been due, not to the presence of fluid, but to that of the testis itself in a soft and healthy condition, held forwards by the solid mass of coagulated blood in which it was imbedded. The absorption of the blood proceeded but very slowly, and it was not until Feb. 1, that it was sufficiently accomplished to permit of the patient being discharged, and even at that time there remained considerable thickening of the cord, the other parts being left, however, in a perfectly normal state.

CASE II. *Probable Injury to the Groin.—Large Extravasation of blood into the structures of the cord.—Treatment by mercury.—Slow absorption of the effused blood.* Under the care of Mr. SOLLY.—The following case, although similar to the preceding in its main features, was much complicated, as to diagnosis, by the history of an old standing hernia on the same side:

John Grady, a potman, aged 30, was admitted on Dec. 9, 1852. In the right side of his scrotum, the testicle and cord were concealed by an elongated swelling, the size of a fist, which extended from the most depending part of the scrotum into the inguinal canal. It was of smooth, tense and solid feeling, and the skin might easily be pinched up from it. No impulse was communicated to it by coughing. It was very difficult to arrive at a correct history of its appearance. On one occasion the patient confessed to having a few days before admission been so drunk that he did not know what happened to him, but this he afterwards strenuously denied. He had seven years ago suffered from a hernia on the same side, which was easily returned, and for which he wore a truss two years, when it appeared to be cured, and never again troubled him. The present swelling had, he stated, formed slowly during several days, *commencing from above.* During the first day he suffered much from pain and vomiting, but these symptoms soon subsided. As no indications of strangulated hernia were now present, Mr. Solly did not deem it advisable to perform any exploratory operation. He ordered the man to be confined to bed, to have twelve leeches applied to the scrotum, and to take a pill containing two grains of calomel and half a grain of opium every night and morning. On the following day the leeches were repeated, with the effect of much diminishing the inflammation of the part. The pills having, after five days' employment, induced salivation, were then discontinued. After the lapse of about a fortnight, there was found in the lowest part of the swelling just such a softened spot as we have noticed in the foregoing case, and suspicions were strongly excited of the existence of fluid. As absorption was going on very slowly, Mr. Solly ordered the whole affected part to be enveloped in lint spread with mercurial ointment, under the influence of which a slight ptyalism again resulted, and the swelling continued to diminish, from below upwards. It soon became evident that the soft part which had been felt, was due to the presence of the testis, as in the former instance.

The man left the hospital on Feb. 5, the lower half of the swelling having very nearly vanished, but the cord remaining extremely thick in the upper part and inguinal region.

There can, we think, be no hesitation as to the propriety of considering both the preceding cases as instances of the extravasation of blood within the fascia of the cord, consequent on the rupture either of the spermatic artery or of some of the veins composing the spermatic plexus.

The limitation of the swelling to one side of the scrotum, its rounded outline, pyriform shape, and non-attachment to the integument, all tend to prove that the blood was not free in the scrotal cellular tissue, but confined within some deeper membraneous tunic. The circumstance that the testicle was, in the first stage in each case, completely buried in the mass, is easily explicable by reference to the fact, that the infundibuliform fascia invests not only the cord and epidymis, but also the exterior of the tunica vaginalis: with the latter, however, its connexion is much more close than with other parts; and the small quantity of intervening cellular tissue accounts for the rapidity with which the effused blood (probably very small in quantity at first) became absorbed from that situation. Should extravasated blood, when in large quantity, as in the present cases, be evacuated by incisions? We have seen that its removal by absorption is a very tedious process, that it occupied in one case two months, and in the other six weeks (without being in either complete at the time of discharge;) yet taking all circumstances into account, we are convinced that it is one which should not be interfered with. All surgical authorities are opposed to the opening of collections of blood as a principle; and as it regards this situation in particular, there are on record some instructive cases. Through a mistaken diagnosis, *misled partly by the apparent fluctuation of the testicle below,* Mr. Freke was in one case induced to lay open the tumor, a large quantity of blood escaped, and the man ultimately did well. In a case which occurred to Mr. Pott, however, it was, after the incision, found impossible to discover the bleeding vessel, and castration was performed, on account of the alarming hemorrhage which ensued. Now, although we fully concur with the remark made in Mr. Curling's excellent work on the Testis, in allusion to the latter case, that "modern surgeons will not be inclined to admit that castration was the only remedy," yet the bare risk of such an expedient being necessitated, seems quite to overbalance the anticipated advantage in point of time.—*Lond. Med. Times & Gazette.*

